# Effects of anthropogenic sound on digging behavior, metabolism, Ca^2+^/Mg^2+^ ATPase activity, and metabolism-related gene expression of the bivalve *Sinonovacula constricta*

**DOI:** 10.1038/srep24266

**Published:** 2016-04-11

**Authors:** Chao Peng, Xinguo Zhao, Saixi Liu, Wei Shi, Yu Han, Cheng Guo, Jingang Jiang, Haibo Wan, Tiedong Shen, Guangxu Liu

**Affiliations:** 1 College of Animal Sciences, Zhejiang University, Hangzhou, P.R. China; 2 Ocean College, Zhejiang University, Hangzhou, P.R. China; 3 Applied Acoustic Institute of Hangzhou, Hangzhou, P.R. China

## Abstract

Anthropogenic sound has increased significantly in the past decade. However, only a few studies to date have investigated its effects on marine bivalves, with little known about the underlying physiological and molecular mechanisms. In the present study, the effects of different types, frequencies, and intensities of anthropogenic sounds on the digging behavior of razor clams (*Sinonovacula constricta*) were investigated. The results showed that variations in sound intensity induced deeper digging. Furthermore, anthropogenic sound exposure led to an alteration in the O:N ratios and the expression of ten metabolism-related genes from the glycolysis, fatty acid biosynthesis, tryptophan metabolism, and Tricarboxylic Acid Cycle (TCA cycle) pathways. Expression of all genes under investigation was induced upon exposure to anthropogenic sound at ~80 dB re 1 μPa and repressed at ~100 dB re 1 μPa sound. In addition, the activity of Ca^2+^/Mg^2+^-ATPase in the feet tissues, which is directly related to muscular contraction and subsequently to digging behavior, was also found to be affected by anthropogenic sound intensity. The findings suggest that sound may be perceived by bivalves as changes in the water particle motion and lead to the subsequent reactions detected in razor clams.

Anthropogenic sounds generated by large cargo ships, seismic surveys, drilling and pile drivers, recreational holiday boats, and offshore constructions have significantly increased in the past decade^1^,^2^,^3^,^4^,^5^,^6^, creating a new type of pollution—ocean noise—which affects marine organisms inhabiting coastal areas and the open sea. In comparison to other environmental pollutions, ocean noise is regarded as more hazardous, owing to its universal and uncontrollable characteristics^7^. Since marine organisms utilize sound intensively to obtain information about their surrounding environments, noise pollution poses considerable hindrances to intra-specific and inter-specific communication, orientation, mate searching, predator/prey detection, and object identification through acoustic perception^8^,^9^,^10^,^11^. To date, a wide variety of marine organisms, including invertebrates, fish, and marine mammals has been reported to be affected by anthropogenic sound^12^,^13^,^14^. Moreover, the impact of sounds on marine organisms has been shown to be variable and species-specific. Anthropogenic sound may result in no effect^15^, temporary/permanent shifts in hearing threshold or auditory masking^5^,^8^,^16^,^17^, individual and social behavior alteration^9^,^18^,^19^,^20^, physiological damage to sensory organs^10^,^21^,^22^,^23^, altering body metabolisms^4^,^24^,^25^, hampering embryogenesis^12^ and even immediate death^26^. Although numerous studies have investigated the effects of anthropogenic sound on marine organisms such as fish and mammals, to the best of our knowledge, only a few have been performed on mollusk species^20^,^21^,^22^, especially marine bivalves^12^, putting a constraint on a more comprehensive understanding of ocean noise pollution. Furthermore, the underlying molecular mechanisms remain unclear for many organisms, such as bivalves.

The razor clam *Sinonovacula constricta* (Lamarck, 1818) is an important aquaculture species that is widely distributed in the estuaries and intertidal zones of China, Japan, and Korea^27^,^28^,^29^. As a typical bottom-burrowing bivalve, the slender-shaped razor clam has a muscular foot, a light-weight shell, and two vimineous siphons that are adapted to a digging lifestyle. Digging and hiding are crucial in helping *S. constricta* to avoid predators and harsh environments. Furthermore, alterations in digging behavior pose a significant impact on the razor clam aquaculture industry since an increase in digging depth of the organism will increase harvest workload. Digging behavior is the direct result of foot muscular movement and is closely linked to the biological energy supply of the muscle^30^. The supply of movement energy is mainly provided through the glycolysis pathway and TCA cycle^30^. However, the present knowledge of *S. constricta* is mainly limited to its ecological traits^31^,^32^,^33^, nutritional content^34^, cultivation techniques^35^,^36^, and genetic background^37^,^38^,^39^,^40^,^41^,^42^. The effects of anthropogenic sounds on the digging behavior and the underlying mechanisms remain elusive.

The main objectives of the present study were to: 1) explore if anthropogenic sounds affect the digging behavior of the razor clam; 2) find out whether sound type, frequency, and/or intensity contribute to the changes detected in digging behavior; 3) investigate the effects of anthropogenic sounds on the metabolism of the razor clam; and 4) identify genes associated with the altered digging behavior in response to sound changes.

## Results

### Acoustic conditions in seawater

As shown in Table 1, due to sound reflection and mud absorption^43^, the intensities of experimental sounds were significantly lower in the sediment than that at the interface between seawater and the sediment. With the same sound source, there was no significant difference found in the sound intensity at different distances (5, 15, 25cm), taken from the center of the concentric circle vertically below the sound source.

### Effects of sound type, frequency, and intensity on the digging behavior of razor clams

**Figure 1:**
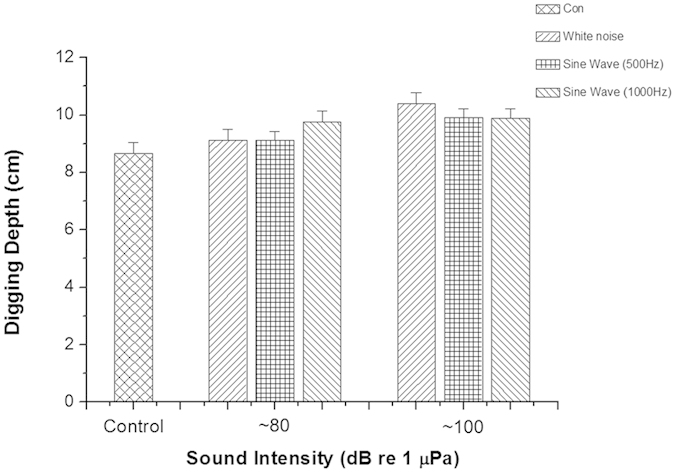
Digging results for different treatment groups. The digging depths (mean ± SE) of the razor clams exposed to anthropogenic input sounds of various types, frequencies, and intensities in an experimental period of 24 hours.

As shown in Fig. 1 and Table 2, the digging depths of the razor clams exposed to the 500 Hz sine wave (aeration background noise plus anthropogenic sine wave sound input) were not significantly different from those exposed to 1000 Hz of the same sound type. *P* values were 0.21 and 0.96 from the ANOVA for the ~80 dB re 1 μPa and ~100 dB re 1 μPa trials, respectively. Similarly, the digging behavior was not affected much by various anthropogenic input sound types, where no significant difference in digging depths was detected between the “white noise” (broad frequency sound generated with anthropogenic white noise input) and sine wave sound input groups, with trials carried out at the same underwater sound intensity. Interestingly, the three sound intensities tested led to a significant difference in the digging depths (*p* < 0.05) of the clams. Generally, digging depth increased with underwater sound intensity. The digging depths of razor clams exposed to anthropogenic sound of “white noise”, 500 Hz sine wave, and 1000 Hz sine wave at ~80 dB re 1 μPa were 1.05, 1.05, and 1.13 times deeper respectively than those exposed to the control (~60 dB re 1 μPa of ambient aeration noise). When the underwater sound intensity was increased to ~100 dB re 1 μPa, the digging depths of razor clams exposed to anthropogenic sound of “white noise”, 500 Hz sine wave, and 1000 Hz sine wave were 1.20, 1.15, and 1.14 times deeper, respectively, than those exposed to the control. These results indicated that the digging behavior of the razor clams was significantly affected by underwater sound intensity.

### Effects of anthropogenic sound exposures on oxygen consumption, ammonia excretion, O:N ratio, and Ca^2+^/Mg^2+^ ATPase activity of razor clams

The oxygen consumption rates, ammonium excretion rates, and O:N ratios of razor clams exposed to the ambient aeration sound (control) or to ~80 dB re 1 μPa or ~100 dB re 1 μPa of “white noise” for one week were listed in Table 3. Results from ANOVA showed that while both the rates of oxygen consumption and ammonia excretion were not significantly different among trials, the O:N ratios were significantly affected by underwater sound intensity. The highest and lowest O:N ratios were detected in the samples exposed to the ~80 dB re 1 μPa and ~100 dB re 1 μPa of underwater sound respectively.

**Figure 2:**
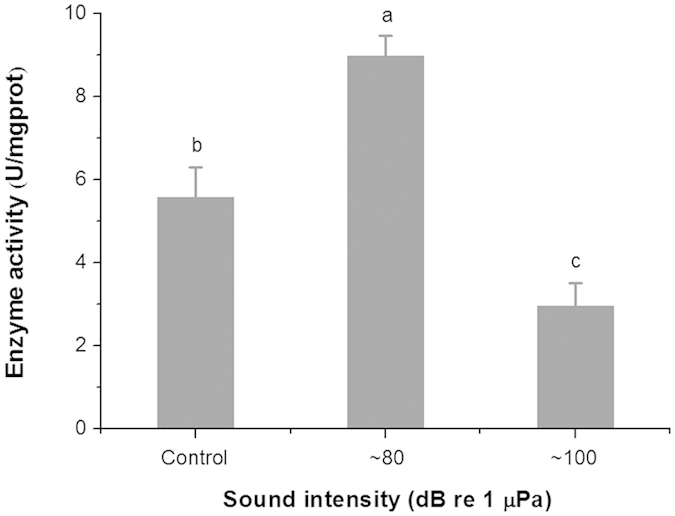
Enzyme activities for different sound intensity groups. The activities of the Ca^2+^/Mg^2+^-ATPase (mean ± SE) of the razor clams after one week of exposure to the ambient sound (control) or to ~80 dB re 1 μPa or ~100 dB re 1 μPa of underwater sounds (different superscript indicates significant difference between trials by Tukey’s test).

As shown in Fig. 2, the intensity of exposed sound exerted a significant effect on the activity of Ca^2+^/Mg^2+^-ATPase, with the highest (8.97 ± 0.49 *U/mg* protein) and lowest activity (2.95 ± 0.56 *U/mg* protein) observed in clams exposed to ~80 dB re 1 μPa and ~100 dB re 1 μPa of underwater sound, respectively.

### Effects of sound exposures on the relative expression of metabolic genes

**Figure 3:**
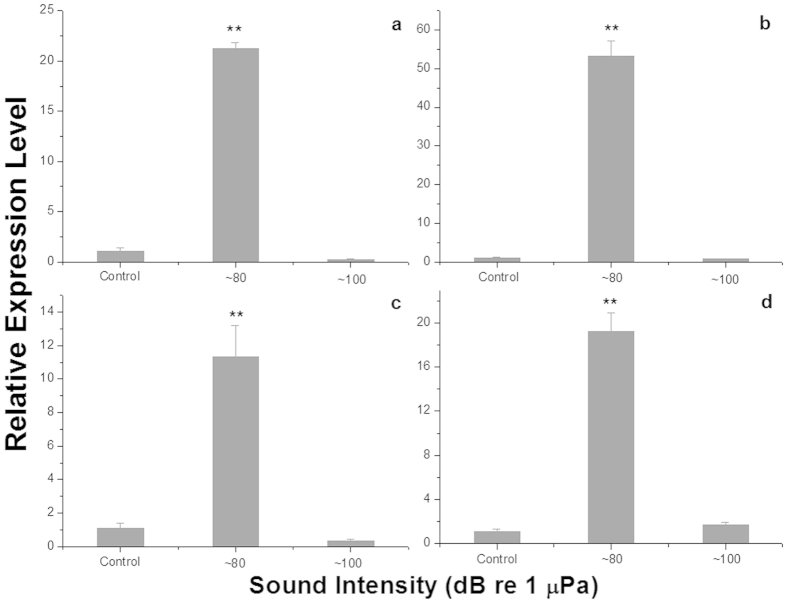
Relative gene expression levels for different sound intensity groups. Relative gene expression levels (mean ± SE) of 6-phosphofructokinase-1 (**a**), pyruvate kinase (**b**), acetyl-CoA carboxylase (**c**), and arylformamidase (**d**) in razor clams exposed to one week of ambient sound or to ~80 dB re 1 μPa or ~100 dB re 1 μPa of underwater sounds (* and **indicate a significant and an extremely significant difference by t-test, respectively, relative to the control of ambient sound).

As shown in Fig. 3a,b, the 6-phosphofructokinase-1 and pyruvate kinase genes, both function in the glycolysis pathway, showed similar relative gene expression patterns. In clams exposed to ~80 dB re 1 μPa of underwater sound, both genes showed significantly higher expression than those of control or exposed to ~100 dB re 1 μPa of sound. Between the groups exposed to quiet ambient sound or ~100 dB re 1 μPa of sound, gene expression was found to be higher in clams exposed to quiet ambient sound, but the difference was not statistically significant.

The relative expression of acetyl-CoA carboxylase and arylformamidase genes in the fatty acid biosynthesis and tryptophan metabolism pathways showed a similar trend (Fig. 3c,d) as well. The only difference was found in a lower relative expression of the arylformamidase gene in clams exposed to the quiet ambient sound, albeit not significant when compared to that in the clams exposed to ~100 dB re 1 μPa of underwater sound.

**Figure 4:**
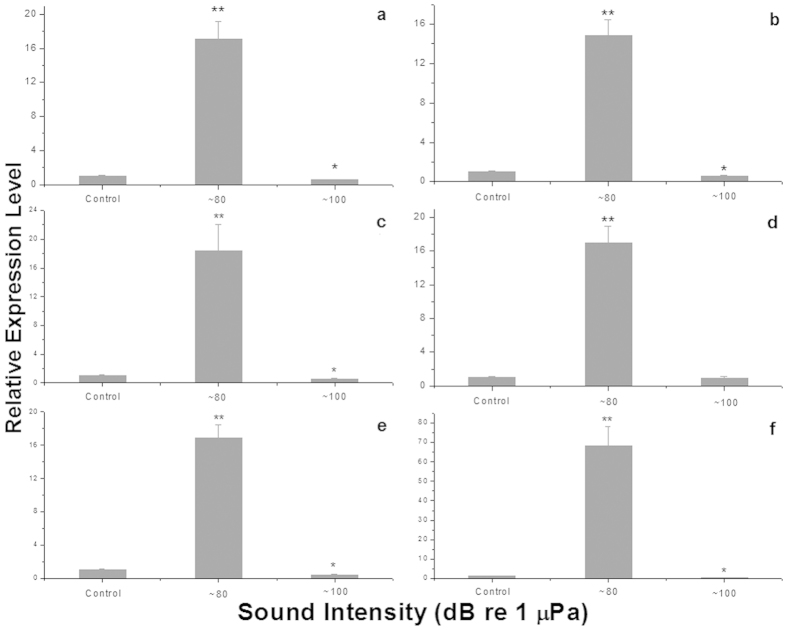
Relative gene expression levels for different sound intensity groups. Relative gene expression levels (mean ± SE) of citrate synthase (**a**), isocitrate dehydrogenase (NAD^+^) (**b**), isocitrate dehydrogenase (NADP^+^) (**c**), oxoglutarate dehydrogenase (E1) (**d**), dihydrolipoamide succinyltransferase (E2) (**e**) and dihydrolipoamide dehydrogenase (E3) (**f**) in razor clams after exposure for one week to ambient sound or to ~80 dB re 1 μPa or ~100 dB re 1 μPa of “white noise” underwater sounds (* and **indicate a significant and an extremely significant difference by t-test, respectively, relative to control of ambient sound).

All six genes in the TCA cycle (Fig. 4) displayed similar relative expression patterns, where highest values were detected in clams exposed to ~80 dB re 1 μPa of sound. Relative gene expression was significantly lower in clams exposed to ~100 dB re 1 μPa of sound than those of the control, with the exception of the oxoglutarate dehydrogenase gene.

## Discussion

Theoretically, in the far field of an acoustic source, the pressure (*p*) and velocity components (*v*) are related as *p* *=* *v* × *z*, where *z* is the parameter that indicates the impedance of the medium^12^. Therefore, with the same sound type and frequency, higher intensity sound, such as the ~80 re 1 μPa and ~100 dB re 1 μPa of underwater sound tested in the present study, will give rise to a more intense particle motion and subsequently lead to a higher sound pressure level in the seawater. Though sensory hearing has not been reported in bivalve species, both the mantle and gills of a bivalve possess sensory palps that are sensitive to environmental disturbances such as water proton movement. In *S. constricta*, in addition to the mantle and gills, foot, exhalent siphon, inhalant siphon, and the palps around these organs are sensitive to the motion of surrounding water as well. Few studies have suggested that sound may exert influences on the embryonic development and settlement of marine bivalves^12^,^44^,^45^. With field experiments, it has been shown that the addition of replayed habitat-related sounds significantly increased the settlement of free-swimming larvae of the eastern oyster (*Crassostrea virginica*), compared to no-sound controls^44^,^45^. Whereas a laboratory study carried out in New Zealand scallop (*Pecten novaezelandiae*) has shown that the noise generated by a seismic air gun significantly increased the percentage of body malformations and retarded the growth and development of the larvae^12^, probably owing to the increase in sound pressure. Similarly, the variations detected in the present study in digging behavior, metabolism, and expression of metabolic genes in response to noise exposure at different intensities could attribute to the changes in water particle motion as well.

The digging behavior of the razor clam, a bottom burrower that lives in the intertidal zone, is an adaptation to its surroundings and is closely related to water movement. With alterations in the ebb and flow, the razor clam exhibits corresponding digging or emerging behaviors. Importantly, the digging behavior of the razor clam is closely related to its metabolism status. On the one hand, as a result of muscular movement of the foot, digging, or emerging behavior is an energy-consuming process that relies on the energy produced through pathways such as glycolysis and the TCA cycle. On the other hand, digging behavior is associated with active feeding because shallow digging increases the contact with oxygen and food supplies in seawater, albeit accompanied by an increased risk of encountering predators and environmental stressors.

In the present study, exposure to the intensified ~100 dB re 1 μPa of underwater sound induced an avoidance response in the razor clams, resulting in a significantly more active digging activity. Similar to other environmental stressors such as unfavorable temperature, salinity, and pH, exposure to ~100 dB re 1 μPa of underwater sound also led to a significant decrease in the O:N ratio, indicating a higher rate of protein catabolism relative to lipid and carbohydrate catabolism^31^,^32^. This observation may be due to the reduction in oxygen availability associated with deeper digging, a behavior that may occur in response to stress, as previously reported in other species^46^,^47^. When razor clams were exposed to the intensified ~100 dB re 1 μPa of underwater sound, the expression of all tested genes from the glycolysis^48^, fatty acid biosynthesis^49^, tryptophan metabolism^50^,^51^, and TCA cycle^52^,^53^ pathways were repressed, suggesting a slowing of metabolic activity as the individual retreat deeper into the mud. To give a brief summary, the intensified water particle movement brought about by ~100 dB re 1 μPa of underwater sound rendered the clams digging deeper into the mud and entering an inactive state, as a means to avoid environmental disturbance.

Unlike the ambient aeration sound from indoor cultivation and the ~100 dB re 1 μPa of underwater sound, the ~80 dB re 1 μPa of sound rendered the clams more active, as indicated by the significantly higher O:N ratio^46^,^47^,^54^ and increased expression of genes from the glycolysis^48^, fatty acid biosynthesis^49^, tryptophan metabolism^50^,^51^, and TCA cycle pathways^52^,^53^. Anthropogenic sound at ~80 dB re 1 μPa is comparable to the natural ambient sound level in the intertidal zone when the bottom substrates are covered by seawater^55^,^56^. Therefore, exposure to ~80 dB re 1 μPa of broad frequency underwater sound probably created a similar water particle movement condition indicating high food and oxygen availability for razor clams and subsequently induced shallow digging behavior for feeding. The expression of genes involved in glycolysis and the TCA cycle in clams exposed to ~80 dB re 1 μPa of underwater sound was in accordance with the O:N data obtained, which was significantly higher than those in clams exposed to ambient control and ~100 dB re 1 μPa of intensified underwater sound. Upon activation by ~80 dB re 1 μPa of underwater sound, the clams exhibited more catabolism of carbohydrates and lipids relative to protein to meet the new energy demands associated with the induced active feeding behavior. Similarly, as tryptophan catabolism will produce a series of biologically active substances that play important roles in physiological metabolism^51^,^57^, the arylformamidase gene in the tryptophan catabolism pathway was found to be activated. The increase in active feeding behavior also triggered the storage of energy as fatty acids, which was indicated by a significantly higher expression of the acetyl-CoA carboxylase gene from the fatty acid synthesis pathway^49^.

Less digging depth was observed when razor clams were exposed to the relatively quiet ambient aeration sound. This indoor cultivation sound was quieter than the natural ambient sound in the intertidal zone. As discussed above, sound is probably perceived by bivalve species as a variation in water particle movement, which delivers information about the magnification of flow or tides. Therefore, less digging was probably exhibited because the quiet ambient sound was regarded as “risk-free” by the individual razor clams. Moreover, the mild water particle movement driven by the quiet ambient sound neither activated nor inhibited the metabolism of the razor clams, which was observed with the ~80 dB re 1 μPa and ~100 dB re 1 μPa underwater sound treatments.

The digging and/or emerging behaviors of bottom-burrowing bivalves are directly controlled by foot muscular movement through an energy-consuming process^30^. The digging behavior has been reported to be Mg^2+^-concentration dependent in the hard clam *Meretrix lusoria*^58^,^59^. The Ca^2+^/Mg^2+^-ATPase is an Mg^2+^-dependent enzyme and directly related to muscular contraction^60^,^61^,^62^,^63^,^64^; therefore, the status of Ca^2+^/Mg^2+^-ATPase in the foot can be used as an indicator of digging and/or emerging activities. In the present study, the highest enzyme activity of the Ca^2+^/Mg^2+^-ATPase was found in razor clams exposed to ~80 dB re 1 μPa of underwater sound when active feeding was induced. The high enzyme activity could be accounted by the increase in foot movement during feeding. Though with deeper digs, exposure to the ~100 dB re 1 μPa of underwater sound led to the lowest activity of the Ca^2+^/Mg^2+^-ATPase. As the clams has already dug into the substrate and entered an inactive state to avoid the disturbance before the sampling time-point, the low enzyme activity reflected the resting condition of the individuals at the time of sampling. Similarly, the clams were neither activated nor inhibited by exposure to the ambient sound, with moderate Ca^2+^/Mg^2+^-ATPase activity found in samples under these conditions.

## Methods

### Animal collection and maintenance

Adult razor clams were collected from Yueqing Bay (28.28°N and 121.11°E), Wenzhou, China, before the spawning season from late May to early July of 2014. Once transported to the lab, the clams were acclimated in a 2000 L tank with 500 L aerated flowing seawater (temperature 23.9 ± 1.0 °C, pH 7.95 ± 0.40, salinity 20.0 ± 0.5%, dissolved oxygen 8.01 ± 0.3 mg/L, and total alkalinity 1.91 ± 0.40 mmol/L) for a week before the commencement of experiment. Clams were fed with microalgae *Platymonas subcordiformis* twice a day, at 8:00 a.m. and 8:00 p.m. Healthy individuals with no shell damage and of regular size (shell length at 5.3 ± 1.1 cm) were used for the experiments.

### Sound treatment and record

**Figure 5:**
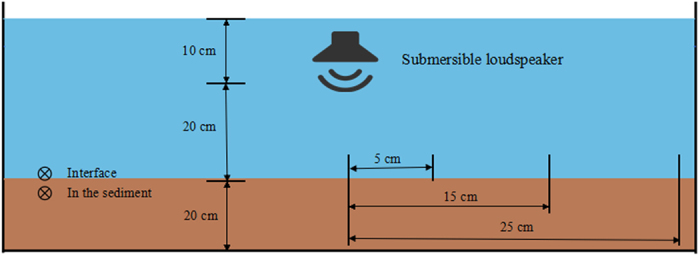
Schematic of the measurement of acoustic conditions in seawater. The hydrophone was deployed in the sediment (the hydrophone was completely buried in the mud) and at the interface between seawater and sediment at 5 cm, 15 cm, 25 cm away from the center of concentric circle that is vertically below the submersible loudspeaker.

Referring to preliminary survey results and published literatures, underwater sound levels of ~80 dB re 1 μPa and ~100 dB re 1 μPa were selected to simulate normal sound level in the intertidal zone when covered by tide and under situations of intensive anthropogenic sound, respectively^55^,^56^. An ambient aeration sound level of the culture system without any addition of anthropogenic sound input was used as control. To find out the factor of the anthropogenic sound affecting digging behavior, the various combinations of two types of sound-input signals (white noise and sine wave) and two frequencies of sine wave sound (500 Hz and 1000 Hz) were tested. In total, the following seven sound exposures with basal ambient aeration noise were investigated: 1) no additional anthropogenic sound (control), 2) ~80 dB re 1 μPa of “white noise” , 3) ~100 dB re 1 μPa of “white noise”, 4) ~80 dB re 1 μPa 500 Hz sine wave, 5) ~100 dB re 1 μPa 500 Hz sine wave, 6) 80 dB re 1 μPa 1000 Hz sine wave, and 7) 100 dB re 1 μPa 1000 Hz sine wave. Experiments were carried out in 160 L plastic buckets containing 20 cm of mud (with particle diameters of 7.34 ± 0.76 μm) covered by 30 cm deep-sand-filtered seawater. Sound broadcast systems is consisted of an submersible loudspeaker (UW-30, Electro-Voice^®^; South Bend, Indiana, USA; frequency response 0.1–10 kHz; impedance 8 ohms; power-handling capacity 30 watts; operate depth for optimum efficiency at no more than 1.2 m) connected to a power amplifier player (PA-1050, JKA^®^; Jinan, Shandong, China; power-handling capacity 50 watts), coupled with a computer with sound-producing software (MyToneTest). The submersible loudspeaker was suspended at 10 cm under water surface to generate the corresponding experimental sound effect. The acoustic conditions in seawater were monitored using acoustics recording units that consisted of a bioacoustics recorder (Song Meter SM2+, Wildlife Acoustics^®^; Concord, Massachusetts, USA; 96 kHz sampling rate, 16 bit, zero recorder gain), and a calibrated omni-directional hydrophone (HIT-96-MIN, High Tech^®^; Long Beach, Mississippi, USA; flat frequency response 0.02–30 kHz, sensitivity −164 dB re 1 V/μPa). The hydrophone was deployed in the sediment (the hydrophone was completely buried in the mud) and at the interface between seawater and sediment at 5 cm, 15 cm, 25 cm away from the center of the concentric circle located vertically below the submersible loudspeaker (Fig. 5).

Acoustic data were analyzed by Soundscape Analysis Software SACS V1.0 (Register number: 2014SR216788) in MATLAB R2013a. The spectral density measurement with bandwidth of 86 Hz was applied to calculate the sound pressure levels of the control and experimental trials and the mean of sound intensity was subsequently obtained by taking the average of each 30 seconds acoustic data. In this calculation method, the sound signals in a very short period of time (10~30 ms) were deemed as smooth and underwent Fourier transformation as following.

Let *x*(*n*) be an acoustic signal defined for all *n* (*n* is the number of sample points in time number) and 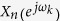 be the short-time Fourier transformation of *x*(*n*) evaluated at time *n* and frequency *ω*_*k*_. Following the method described by Ronald^65^ and Jont^66^, the short-time Fourier transformation was conducted through equation (1).


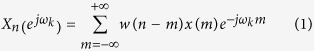


The discrete time Fourier transformation (DTFT) of *x*(*n*) was then obtained via equation (2), in which, *m* is the time serial number which synchronizes with *n* and *N* is the length of given acoustic signal.


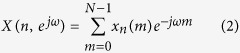


The discrete Fourier transformation (DFT) was then estimated using equation (3), where 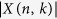 is the short-time magnitude of spectra.


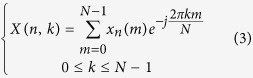


The power spectral density at time *m* was calculated through equation (4).


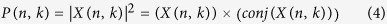


The sound pressure level at each bandwidth was subsequently measured via equation (5), where *P*(*k*) is short-time Fourier spectral density at center frequency and *P*_*0*_ is the reference pressure (1μpa in water).


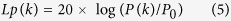


The sound pressure level was calculated through equation (6).


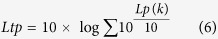


Since the frequency bandwidth is determined by the signal sampling rate and the parameter n of the Fourier transformation, the bandwidth analyzed in the present study is therefore 11025 Hz/128 = 86 Hz (the frequency range after Fourier transformation and n are 0~11025 Hz and 128, respectively).

### Digging behavior trials

Three replicates, each containing 40 experimental individuals, were performed for each trial. A fishing line (0.18 mm in diameter and 1.5 m in length), with a label identifying the clam, was attached with waterproof glue to the shell of each razor clam in a longitudinal direction to facilitate estimations of the digging depth. Upon a gentle straight pull, the change in length of the attached fishing line before and after experiment was defined as the digging depth. With preliminary experiments indicating that all digging behavior of the razor clams was completed within 24 hours after upload onto the bucket, an experimental period of 24 hours was monitored for all the digging behavior trials. The seawater of each trial was aerated throughout the entire experiment, serving as the main ambient sound.

### Oxygen consumption rate, ammonia excretion rate, O:N ratio and Ca^2+^/Mg^2+^ ATPase activity assays

According to the results obtained in the digging behavior trials, digging behavior was mainly affected by sound intensity. Therefore, the effects of underwater sounds with anthropogenic “white noise” input at ~80 dB re 1 μPa and ~100 dB re 1 μPa on oxygen consumption rate, ammonia excretion rate, and Ca^2+^/Mg^2+^-ATPase activity were investigated in the present study.

Apart from exposure timings and fishing line attachment, similar methods described in Digging behavior trials were carried out for the sound exposure experiments. During the one-week exposure, one-half of the seawater volume was replaced daily with fresh filtered seawater, and the clams were fed with microalgae *P. subcordiformis* at 8:00 a.m. and 8:00 p.m every day. Upon introduction to the ambient sound of control, ~80 dB re 1 μPa or ~100 dB re 1 μPa of underwater sounds for a week, individual razor clams were used to analyze oxygen consumption, ammonia excretion, and Ca^2+^/Mg^2+^-ATPase activity.

Twenty-five individuals from each trial were divided equally into 5 respiratory chambers (2 L) filled with seawater. In total, each sound level tested consisted of 5 replicates containing 5 individuals in each chamber, and a blank trial was conducted with no individual assigned. After buffering the clams in still water for 1 hour, the oxygen consumption rate and the ammonia excretion rate were analyzed. To obtain the oxygen consumption rate, the dissolved oxygen concentrations before and after the experiment were determined by an oxygen meter (Multi 3410 SET4, WTW, Germany). To obtain the ammonia excretion rate, seawater ammonia concentrations before and after the experiment were measured using the standard indophenol blue photometric method^67^. After measurement, the soft tissue of each individual was peeled off carefully with a scalpel and then dehydrated in an 80 °C oven for 10 days. Dry weights of soft tissues were estimated using a Sartorius electronic balance (BSA2245). The oxygen consumption and ammonia excretion rates were calculated using the equation (7):


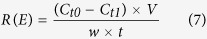


where *R(E)* is the oxygen consumption (or ammonium excretion) rate, *t*_*0*_ and *t*_*1*_ represent oxygen consumption (or ammonium excretion) before and after the experiment, *t* is the total respiration (or excretion) time, *w* is the dry weight of the soft tissues, and *V* is the volume of the respiratory (or excretion) chamber. The atomic ratio of oxygen to nitrogen (O:N) was obtained by dividing the oxygen consumption rate by the ammonia excretion rate.

After one week of sound exposure, another 5 individuals of each experimental trial were used to carry out the Ca^2+^/Mg^2+^-ATPase activity assay. Once carefully peeled off on ice, the foot of each razor clam was used in enzyme activity analysis, following the protocol of the Minim ATP enzyme test kit (Ca^2+^/Mg^2+^ ATPase) from Nanjing Jiancheng Bioengineering Institute^®^. The enzyme Ca^2+^/Mg^2+^ ATPase decomposes ATP into ADP and inorganic phosphate. The concentrations of the inorganic phosphate produced from ATP hydrolysis were determined using a spectrophotometer (UV-2100, Shanghai Jinghua Instruments) at 636 nm. The Ca^2+^/Mg^2+^-ATPase activity was then estimated according to the equation (8). One Ca^2+^/Mg^2+^-ATPase activity unit was defined as the amount of enzyme decomposing 1 μmol ATP per milligram tissue protein per hour.


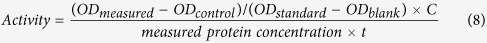


where *OD_measured_, OD_control_, OD_standard_*, and *OD_blank_* indicate the OD values for the tested sample, control, standard sample, and the blank respectively; *C* indicates the concentration of the standard protein; and *t* indicates the reaction time.

### Quantitative real time PCR of metabolic genes

After being exposed to the ambient control, ~80 dB re 1 μPa or ~100 dB re 1 μPa underwater sounds for a week, 5 razor clams from each exposure trial were dissected on ice. The foot of each individual was carefully peeled off with a scalpel and immediately frozen in liquid nitrogen for later use in real-time PCR analysis. Total RNA from different groups was extracted using TRIpure Reagent (Aidlab Biotechnologies Co., Ltd) according to the manufacturer’s instructions. After validating total RNA quality, cDNA was synthesized with an M-MLV First Strand Kit (Invitrogen^®^) and stored at −80 °C.

Based on previous transcriptomic data, the software Primer Premier 5.0 was used to design real-time PCR primers for 10 genes, which encode for 6-phosphofructokinase-1 (JZ897777) and pyruvate kinase (JZ897768) in the glycolysis pathway; acetyl-CoA carboxylase (JZ897769) in the fatty acid biosynthesis pathway; arylformamidase (JZ897770) in the tryptophan metabolism pathway (tryptophan is the first limiting amino acid in razor clams); and citrate synthase (JZ897771), isocitrate dehydrogenase (both NAD^+^ (JZ897772) and NADP^+^ (JZ897773) dependent types) and α-oxoglutarate dehydrogenase (with all the three parts, E1, E2, and E3, representing oxoglutarate dehydrogenase (JZ897774), dihydrolipoamide succinyltransferase (JZ897775) and dihydrolipoamide dehydrogenase (JZ897776), respectively) in the TCA (citrate cycle) pathway respectively. The 18S ribosomal RNA gene was used as an internal reference^68^. The sequences of all primers were listed in Table 4.

Quantitative PCR was performed in a CFX 96^TM^ Real-Time System. Amplifications were carried out in triplicate in a total volume of 10 μL containing 5 μL SsoFast EvaGreen Supermix (Bio-Rad^®^), 3 μL PCR-grade water, 1 μL cDNA, and 1 μL primers (100 μM). The reaction conditions included an initial denaturation at 95 °C for 5 min; 39 cycles of 95 °C for 20 sec, 61 °C for 20 sec; and 72 °C for 20 sec. After the reaction, the Bio-Rad CFX Manager was used for melting-curve analysis to confirm that a specific PCR product was amplified. The 2^−△△CT^ method was applied to analyze the relative gene expression of target genes.

### Statistics

The intensities of sounds in the sediment and at the interface between seawater and sediment at 5 cm, 15 cm, and 25 cm away from the center of concentric circle were analyzed using a one-way ANOVA. To investigate the effect of sound frequency on the digging depth of *S. constricta*, the digging depths of clams exposed to the sine wave anthropogenic sound input with different noise frequencies and constant intensity (~80 or ~100 dB re 1 μPa) were analyzed using a one-way ANOVA. Similarly, a one-way ANOVA was performed on data of the digging depths of razor clams exposed to different types (white noise versus sine wave anthropogenic sound input) and intensities (ambient control, ~80, or ~100 dB re 1 μPa) of sounds to estimate the effects of sound types and intensities on the digging behavior of *S. constricta*. One-way ANOVA followed by Tukey’s post hoc test was conducted to compare the oxygen consumption rates, ammonia excretion rates, O:N ratios, and Ca^2+^/Mg^2+^-ATPase activities among experimental trials. The expression level of each target gene was compared against the control using t-test. All statistics were performed with the statistical package “R” 69,70, and a *p*-value <0.05 was accepted as statistically significant.

## Additional Information

**How to cite this article**: Peng, C. *et al*. Effects of anthropogenic sound on digging behavior, metabolism, Ca^2+^/Mg^2+^ ATPase activity, and metabolism-related gene expression of the bivalve *Sinonovacula constricta. Sci. Rep.* **6**, 24266; doi: 10.1038/srep24266 (2016).

## Figures and Tables

**Table 1:** The acoustic conditions in the experimental setup in the sediment and at the interface between seawater and sediment (data were presented as means and 1st quartile to 3rd quartile of the data in the parentheses, and different superscripts indicate significant differences between trials by Tukey’s test).

**Sound level measured in air**	**Hydrophone position in the experimental setup**	**Underwater sound intensities at distances to the center of the concentric circle of the sediment (dB re 1 μPa)**		
		**5 cm**	**15 cm**	**25 cm**
Control ~50dB re 20 μPa	Interface between water and sediment	63.19^a^ (61.91~64.41)	62.22^a^ (60.96~63.42)	61.67^a^ (60.43~62.86)
	In the sediment	56.13^b^ (55.01~57.40)	55.51^b^ (53.91~57.68)	54.71^b^ (53.12~55.76)
1000Hz~100dB re 20 μPa	Interface between water and sediment	95.55^a^ (86.79~101.54)	94.74^a^ (85.71~~100.89)	93.62^a^ (83.86~100.60)
	In the sediment	87.35^b^ (78.21~94.41)	85.63^b^ (77.67~89.86)	86.93^b^ (78.45~92.32)
1000Hz~80dB re 20 μPa	Interface between water and sediment	87.09^a^ (82.59~88.49)	85.43^a^ (82.08~86.97)	82.15^a^ (79.91~82.64)
	In the sediment	77.92^b^ (76.54~79.44)	77.6504^b^ (76.28~79.21)	77.8985^b^ (76.38~79.11)
500Hz~100dB re 20 μPa	Interface between water and sediment	100.87^a^ (92.77~107.05)	100.16^a^ (92.19~106.54)	96.36^a^ (89.04~103.39)
	In the sediment	85.30^b^ (79.12~89.49)	84.94^b^ (78.91~89.47)	83.5212^b^ (78.55~85.99)
500Hz~80dB re 20 μPa	Interface between water and sediment	86.36^a^ (84.09~86.56)	82.19^a^ (80.29~82.93)	82.69^a^ (79.09~84.48)
	In the sediment	77.48^b^ (75.91~78.92)	77.46^b^ (75.94~78.95)	78.59^b^ (77.28~79.16)
White noise ~100dB re 20 μPa	Interface between water and sediment	103.25^a^ (94.96~109.57)	102.45^a^ (94.30~108.97)	102.21^a^ (94.45~108.67)
	In the sediment	88.82^b^ (82.34~93.18)	88.34^b^ (81.94~93.18)	87.86^b^ (81.49~92.17)
White noise ~80dB re 20 μPa	Interface between water and sediment	92.20^a^ (90.34~96.73)	91.69^a^ (89.84~96.19)	91.17^a^ (89.33~95.65)
	In the sediment	83.09^b^ (81.42~84.64)	84.03^b^ (82.33~85.59)	84.96^b^ (83.24~85.78)

**Table 2:** Digging depths of razor clams after 24-hours’ exposure to underwater sounds of different types, frequencies, and intensities (ANOVA).

**Comparison purposes**	**df**	**SS**	**MS**	***F*****-ratio**	***p*****-value**
**a. Comparisons between different anthropogenic input sound frequencies**					
1) 500 Hz vs 1000 Hz sine wave at ~80 dB re 1 μPa					
Model	1	7.96	7.96	1.63	0.21
Error	76	370.90	4.88		
Total	77	378.86			
2) 500 Hz vs 1000 Hz sine wave at ~100 dB re 1 μPa					
Model	1	0.01	0.01	2.29E-3	0.96
Error	76	306.97	4.04		
Total	77	306.98			
**b. Comparisons between different anthropogenic input sound types**					
1) “White noise” vs 500 Hz sine wave at ~80 dB re 1 μPa					
Model	1	9.35E-4	9.35E-4	1.95E-4	0.99
Error	76	363.45	4.78		
Total	77	363.45			
2) “White noise” vs 500 Hz sine wave at ~100 dB re 1 μPa					
Model	1	4.84	4.84	1.00	0.32
Error	76	366.10	4.82		
Total	77	370.94			
3) “White noise” vs 1000 Hz sine wave at ~80 dB re 1 μPa					
Model	1	7.79	7.79	1.37	0.25
Error	76	432.54	5.69		
Total	77	440.33			
4) “White noise” vs 1000 Hz sine wave at ~100 dB re 1 μPa					
Model	1	5.27	5.27	1.09	0.30
Error	76	367.96	4.84		
Total	77	373.22			
**c. Comparisons among different underwater sound intensities**					
1) Control vs ~80 dB re 1 μPa vs ~100 dB re 1 μPa “white noise”					
Model	2	64.24	32.12	5.52	0.005
Error	114	662.73	5.81		
Total	116	726.97			
2) Control vs ~80 dB re 1 μPa vs ~100 dB re 1 μPa 500Hz sine wave					
Model	2	31.45	15.72	3.32	0.04
Error	114	540.10	4.74		
Total	116	571.55			
3) Control vs ~80 dB re 1 μPa vs ~100 dB re 1 μPa 1000Hz sine wave					
Model	2	35.91	17.95	3.35	0.04
Error	114	611.05	5.36		
Total	116	646.95			

**Table 3:** Oxygen consumption rates, ammonium excretion rates, and O:N ratios of *Sinonovacula constricta* after one week of exposure to the ambient control, ~80 or ~100 dB re 1 μPa of underwater sounds (mean  ±  SE) (Different superscripts indicate significant differences between trials by Tukey’s test).

**Measurements**	**Experimental trials**			***p*****-value**
	**Ambient control**	**~80dB re 1 μPa**	**~100dB re 1 μPa**	
oxygen consumption rate (mg/g·h)	0.88 ± 0.08	1.04 ± 0.06	0.89 ± 0.06	0.21
ammonium excretion rate (μmol/g·h)	8.63 ± 1.02	9.28 ± 0.75	9.99 ± 0.83	0.56
O:N ratio	6.51 ± 0.13^ab^	7.40 ± 0.19^a^	5.72 ± 0.37^b^	0.01

**Table 4:** The primer sequences for the 10 tested genes and the internal reference 18 S rRNA.

**Primers for the genes analyzed**	**Sequence (5′ to 3′)**
6-phosphofructokinase-1-F	GGAATCGTCAGGATAGGTATA
6-phosphofructokinase-1-R	TGCTCTCGTTATTGTTGGA
Pyruvate kinase-F	TCGTGTAATGGCAATAATCG
Pyruvate kinase-R	GTAGAAGCATCGTTCAAGTC
Acetyl-CoA carboxylase-F	TGGATGGCAATGTTGATGA
Acetyl-CoA carboxylase-R	GGCACTGATGGTAGAGAAG
Arylformamidase-F	TTCTTGAAGGCTGGACATT
Arylformamidase-R	GTTAGTGGCAGGCATCTAT
Citrate synthase-F	CAGTTCAGTGCTGCCATA
Citrate synthase-R	CAAGTTACGGTAGATGATAGAC
Isocitrate dehydrogenase(NAD^+^)-F	GCAGGCAAGATGGTATGA
Isocitrate dehydrogenase(NAD^+^)-R	GATGCTGAGATGTCTATGGA
Isocitrate dehydrogenase(NADP^+^)-F	ATGTTGCTAAGGATGTTACC
Isocitrate dehydrogenase(NADP^+^)-R	TTAGGAGATGGACTGTTCTT
Oxoglutarate dehydrogenase-F	GGCATTACAACAGAAGAGAAG
Oxoglutarate dehydrogenase-R	GTAGACACGCTGGAAGATG
Dihydrolipoamide succinyltransferase-F	GCATAGGTCTGGATAGCA
Dihydrolipoamide succinyltransferase-R	CTTGTTGTTCTACCATGTTG
Dihydrolipoamide dehydrogenase-F	ACAGGCTCTGAAGTCACA
Dihydrolipoamide dehydrogenase-R	GCACCAATCACAATCATCTT
18S ribosomal RNA-F	TCGGTTCTATTGCGTTGGTTTT
18S ribosomal RNA-R	CAGTTGGCATCGTTTATGGTCA
